# Comparison of COVID-19 and seasonal influenza under different intensities of non-pharmaceutical interventions and vaccine effectiveness

**DOI:** 10.3389/fpubh.2022.973088

**Published:** 2022-09-27

**Authors:** Yinchang Chen, Zhende Wang, Feng Li, Jingyu Ma, Jie Zhang, Yunpeng Chen, Ting Zhang

**Affiliations:** ^1^Department of Journalism and Communication, School of Media and Law, NingboTech University, Ningbo, China; ^2^School of Public Health, Weifang Medical University, Weifang, China; ^3^Department of General Office, China Health Education Center, Beijing, China; ^4^School of Public Health, Qilu Medical College, Shandong University, Jinan, China; ^5^Department of Immunoprophylaxis, Zhangdian Center for Disease Control and Prevention, Zibo, China; ^6^Bartlett School of Sustainable Construction, University College London, London, United Kingdom; ^7^School of Population Medicine and Public Health, Chinese Academy of Medical Sciences and Peking Union Medical College, Beijing, China

**Keywords:** COVID-19 pandemic, seasonal influenza, non-pharmaceutical interventions, vaccine, transmission dynamics model

## Abstract

**Background:**

The COVID-19 pandemic has lasted more than 2 years, and the global epidemic prevention and control situation remains challenging. Scientific decision-making is of great significance to people's production and life as well as the effectiveness of epidemic prevention and control. Therefore, it is all the more important to explore its patterns and put forward countermeasures for the pandemic of respiratory infections.

**Methods:**

Modeling of epidemiological characteristics was conducted based on COVID-19 and influenza characteristics using improved transmission dynamics models to simulate the number of COVID-19 and influenza infections in different scenarios in a hypothetical city of 100,000 people. By comparing the infections of COVID-19 and influenza in different scenarios, the impact of the effectiveness of vaccination and non-pharmaceutical interventions (NPIs) on disease trends can be calculated. We have divided the NPIs into three levels according to the degree of restriction on social activities (including entertainment venues, conventions, offices, restaurants, public transport, etc.), with social controls becoming progressively stricter from level 1 to level 3.

**Results:**

In the simulated scenario where susceptible individuals were vaccinated with three doses of COVID-19 coronaVac vaccine, the peak number of severe cases was 26.57% lower than that in the unvaccinated scenario, and the peak number of infection cases was reduced by 10.16%. In the scenario with level three NPIs, the peak number of severe cases was reduced by 7.79% and 15.43%, and the peak number of infection cases was reduced by 12.67% and 28.28%, respectively, compared with the scenarios with NPIs intensity of level 2 and level 1. For the influenza, the peak number of severe cases in the scenario where the entire population were vaccinated was 89.85%, lower than that in the unvaccinated scenario, and the peak number of infections dropped by 79.89%.

**Conclusion:**

The effectiveness of COVID-19 coronaVac vaccine for preventing severe outcomes is better than preventing infection; for the prevention and control of influenza, we recommend influenza vaccination as a priority over strict NPIs in the long term.

## Introduction

The COVID-19 pandemic has lasted more than 2 years since its outbreak in 2019. According to the World Health Organization (WHO), as of 3 August 2022, there have been 577,018,226 confirmed cases of COVID-19, including 6,401,046 deaths (1). Despite multiple epidemic waves, the pandemic does not appear to have been effectively controlled. As more and more countries gradually relax their COVID-19 prevention and control policies and opt for a governance model of co-existence with the virus, the development trend of the COVID-19 and its future impact will be more and more worthy of attention. The reason COVID-19 has had such a widespread and dramatic impact is because the SARS-CoV-2 is highly contagious and spread rapidly. There are a large number of asymptomatic infections, which poses challenges to case detection. Fortunately, viral virulence and transmission characteristics can be estimated from existing and previous outbreaks, making it possible to model disease transmission using mathematical methods.

The transmission dynamic model is widely used in the analysis of epidemic trends of infectious diseases. Based on the simulation at different time, we can formulate targeted prevention and control strategies, allocate medical resources scientifically, and maintain the proper operation of the public health system.

Currently, COVID-19 is still a significant public health emergency in China. Therefore, the Chinese government has adopted a “dynamic zero-COVID” policy strategy to minimize the epidemic's peak and delay the time to peak. Today, this strategy still plays an important role in the rapid control of the outbreaks and the prevention of the spread of COVID-19 in China. If the “dynamic zero-COVID” policy is abandoned, it can be predicted that a large number of new cases will emerge in the short term (2). However, we cannot ignore the economic and psychological burdens on Chinese society. Therefore, in the context of the omicron variant being the dominant variant strain, it is necessary to fully understand and explore the new epidemic characteristics of the omicron variant and adopt a better strategy against COVID-19. At the same time, the vaccination against COVID-19 worldwide is continuing, and we expect to use the model to make a preliminary quantitative assessment of the vaccination effectiveness.

In the past century, there have been five pandemics of respiratory infectious diseases, each of which has caused serious infection and mortality in humankind. Among them, the 1918 influenza pandemic infected about a third of the world's population and caused about 50 million deaths worldwide (3). The death toll due to the influenza pandemic of 1957–1958 is estimated at over 1 million (4); the death toll due to the influenza pandemic of 1968–1969 is estimated at 1–4 million (5).

A study showed that NPIs applied to COVID-19 also reduced influenza activity intensity in southern and northern China and the United States by 79.2%, 79.4%, and 67.2%, respectively (6). The prevention and control of COVID-19 pandemic provide an opportunity to study the epidemic patterns and prevention and control strategies of the influenza pandemic. The influenza pandemic is uncertain and inevitable. It is difficult to predict what new subtype will cause the next influenza pandemic, when and where it will occur, and there is even the possibility of the coexistence of influenza and COVID-19 pandemic. Therefore, the exploration of NPIs and the protective effectiveness of vaccines also play a positive role in preventing and controlling the influenza pandemic. This study compares the infection process and scale of COVID-19 and influenza under different scenarios to provide quantitative evidence for countries to optimize prevention and control strategies appropriately.

## Methods

### Formulation of mathematical model

**Figure 1:**
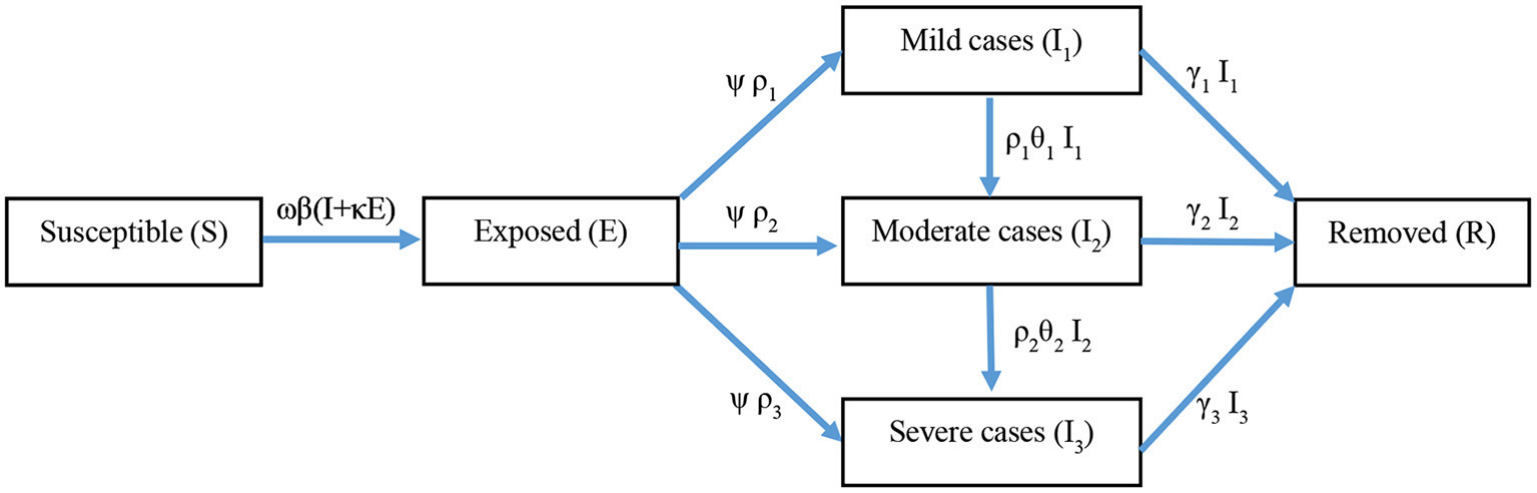
The transmission chain of transmission dynamics model is constructed according to epidemic characteristics of diseases. The SEIR model includes six compartments, i.e., Susceptible (*S*), Exposed (*E*), Mild cases (*I*_1_), Moderate cases (*I*_2_), Severe cases (*I*_3_), and Removed (*R*).

Modeling of etiological and epidemiological characteristics was conducted based on COVID-19 and influenza pandemics using transmission dynamics models to assess the vaccine protection against infection and its disease severity and the impact of non-pharmaceutical interventions (NPIs) on the prevalence intensity of COVID-19 and influenza pandemics. We designed an improved SEIR model to show individuals' transition between compartments based on disease status. Figure 1 shows the primary infectious disease transmission structure of the model. Different NPIs levels, vaccination effectiveness, and transmission patterns, all these factors have been considered in this model. Non-pharmaceutical intervention can prevent the infected rate per contact and can prevent contact rate per unit of time. Based on concepts developed for vaccine efficacy, the immune efficacy generated by infection or vaccination can reduce susceptibility to infection, reduce infectiousness, and reduce pathology. All these factors could change the value of parameters in the model.

The system of differential equations is shown below,


$$\begin{array}{l} \frac{dS}{dt}=-\omega \beta S\left(I+\kappa E\right)/N\\ \frac{dE}{dt}=\omega \beta S\left(I+\kappa E\right)/N-\text{ Ψ }E\\ \frac{dI_{1}}{dt}=\psi \rho_{1}E-\rho_{1}\theta_{1}I_{1}-\gamma_{1}I_{1}\\ \frac{dI_{2}}{dt}=\psi \rho_{2}E+\rho_{1}\theta_{1}I_{1}-\gamma_{2}I_{2}-\rho_{2}\theta_{2}I_{2}\\ \frac{dI_{3}}{dt}=\psi \rho_{3}E+\rho_{2}\theta_{2}I_{2}-\gamma_{3}I_{3}\\ \frac{dR}{dt}=\gamma_{1}I_{1}+\gamma_{2}I_{2}+\gamma_{3}I_{3}\\ N\left(t\right)=S\left(t\right)+E\left(t\right)+I_{1}\left(t\right)+I_{2}\left(t\right)+I_{3}\left(t\right)+R\left(t\right)\\ I\left(t\right)=I_{1}\left(t\right)+I_{2}\left(t\right)+I_{3}\left(t\right)\\ 1=\rho_{1}+\rho_{2}+\rho_{3}. \end{array}$$

### Description of variables and parameters

As we mentioned above, the SEIR model includes six main variables, i.e., Susceptible (*S*), Exposed (*E*), Mild cases (*I*_1_), Moderate cases (*I*_2_), Severe cases (*I*_3_), and Removed (*R*). The relationships between them are linked by specific parameters. The variables and specific parameters in the model are set according to the relevant information, including references and expert suggestions. Details of the variables and parameters are shown in Table 1.

**Table 1:** SEIR model variables and parameters.

**Parameters**	**Description**
*S*	Susceptible population
*E*	Exposed (contagious but not showing symptoms)
*I*_1_	Mild cases (patients with asymptomatic or mild flu-like symptoms such as fever, fatigue, cough, anorexia, malaise, muscle pain, sore throat, dyspnea, nasal congestion, headache)
*I*_2_	Moderate cases (mild or moderate clinical features. Chest imaging showed mild pneumonia manifestation)
*I*_3_	Severe cases (patients who showed severe respiratory failure, required respiratory support, or must be admitted to the ICU)
*r*	Recovered^a^
ω	Control intensity index, indicating the percentage of infected cases reduced by control measures
β	Transmission coefficient, indicating the average number of susceptible people who are infected by one infectious case (including those who are ill and those in the incubation period) in unit time
κ	Infectivity discount coefficient of infected persons in incubation period compared with infected persons with onset
γ_1_, γ_2_, γ_3_	The recovery rate of mild, moderate, and severe/critical cases, respectively, that is, the reciprocal of the recovery period
ρ_1_, ρ_2_, ρ_3_	The composition ratio of mild, moderate, and severe/critical cases, respectively
θ_1_, θ_2_	The rate at which mild cases convert to moderate cases, and moderate cases convert to severe/critical cases
ψ	The rate from infection to onset, namely the reciprocal of the incubation period

a In the SEIR model, the compartment “removed” included recovered and death cases. In this study, we focused more on trends of maximum infections scale, which is closely linked with the health care burden, so that no death cases were involved.

### Scenarios setting

Based on the epidemiological and virological characteristics of the epidemic, 10 different scenarios were constructed to simulate the epidemic curve in a city with a population of 100,000. There were three levels of NPIs included in this study. We assumed that levels 1, 2, and 3 NPIs reduced the effective reproduction number (Rt) by 47%, 55%, and 69%, respectively (7). The vaccination effectiveness in preventing infection of COVID-19 and influenza were set to 33% (after three doses of CoronaVac (0.5 ml given intramuscularly) vaccination) (8) and 50% (9), respectively. Scenarios 1–5 were simulations of COVID-19, and scenarios 6–10 were simulations of influenza, with different parameter combinations for each scenario. The effects of NPIs were not considered in scenarios 1, 2, and 6, 7, which represented the natural epidemic scenario and were used to exclude the difference between NPIs. Scenario 2 was compared with scenario 1 to analyze the epidemic pattern of COVID-19 under different effectiveness of vaccination; scenario 2 was compared with scenario 7 to analyze the epidemic pattern of COVID-19 and influenza after vaccination. In scenarios 3–5/8–10, the effect of NPIs intensity on the epidemic trend was evaluated by simulating the effects of different NPIs intensities on COVID-19 and influenza based on the description and analysis of the time to reach the peak of cases and the maximum number of cases. Finally, scenarios 1–5 were compared with scenarios 6–10 to analyze the effect of NPIs with the same intensity on the epidemic trend of COVID-19 and influenza in the same initial state. See Tables 2 and 3 for more details of all scenarios.

**Table 2:** Scenarios setting.

**Scenarios**	**Disease**	**NPI levels**	**Effectiveness of vaccination**
Scenario 1	COVID-19	No	No
Scenario 2	COVID-19	No	33% for preventing infection (8)
Scenario 3	COVID-19	Level 3	No
Scenario 4	COVID-19	Level 2	No
Scenario 5	COVID-19	Level 1	No
Scenario 6	Influenza	No	No
Scenario 7	Influenza	No	50% for preventing infection (9)
Scenario 8	Influenza	Level 3	No
Scenario 9	Influenza	Level 2	No
Scenario 10	Influenza	Level 1	No

Level 1: NPIs announced or implemented before civil servants work from home (WFH), which usually include tightened social distancing measures in restaurants and indoor leisure facilities, and closure of kindergartens and primary schools. Level 2: NPIs announced or implemented together with civil servants WFH, which often include the closure of most indoor leisure facilities, closure of all schools, no dine-in in restaurants after 9 pm. Level 3: NPIs announced or implemented after civil servants WFH, which include more stringent control measures of restaurants, such as no dine-in after 6 pm or all day (7).

**Table 3:** Parameter combinations of five scenarios of COVID-19.

**Parameters**	**Scenario**					**Setting basis**
	**Scenario 1**	**Scenario 2**	**Scenario 3**	**Scenario 4**	**Scenario 5**	
*S*	1 × 10^5^	1 × 10^5^	1 × 10^5^	1 × 10^5^	1 × 10^5^	Hypothesis
*E*	24	24	63	63	63	I time the incubation period
*I*_1_	5	5	10	10	10	Hypothesis
*I*_2_	1	1	6	6	6	Hypothesis
*I*_3_	1	1	2	2	2	Hypothesis
*I*	7	7	18	18	18	I_1_ + I_2_ + I_3_
*R*	0	0	0	0	0	Hypothesis
Ω	–	–	0.31	0.45	0.53	Reference (7)
β	1.3	1.3 × 0.67	1.3	1.3	1.3	Calculation by experts (8, 10) Reference (8)
κ	0.35	0.35	0.35	0.35	0.35	Reference (2)
γ_1_	1/7	1/7	1/7	1/7	1/7	Reference (11)
γ_2_	1/10	1/10	1/10	1/10	1/10	References (11, 12)
γ_3_	1/19	1/19	1/19	1/19	1/19	Reference (11)
ρ_1_	0.51	0.57	0.51	0.51	0.51	References (13, 14) and calculated according to ρ_2_, ρ_3_
ρ_2_	0.30	0.34	0.30	0.30	0.30	Calculated according to ρ_3_
ρ_3_	0.19	0.09	0.19	0.19	0.19	References (15–17)
θ_1_	1/5.9	1/5.9	1/5.9	1/5.9	1/5.9	Reference (11)
θ_2_	1/8.3	1/8.3	1/8.3	1/8.3	1/8.3	Reference (11)
ψ	1/3.5	1/3.5	1/3.5	1/3.5	1/3.5	Reference (18)

In this study, ρ_1:_ ρ_2_ approximate equal to 5:3, according to the reference (13), the proportion of mild cases is about equal to that of moderate cases, but we take asymptomatic infections [20% (14)] as mild cases, so the proportion of ρ_1_ and ρ_2_ confirmed.

### Statistical analysis

R software version 4.0.5 (the R Foundation for computing) software and deSolve software package were used for modeling and analysis, and Microsoft Office 2013 was used for data cleansing and description.

## Results

### Effects of COVID-19 vaccine—Scenario 1 vs. scenario 2

**Figure 2:**
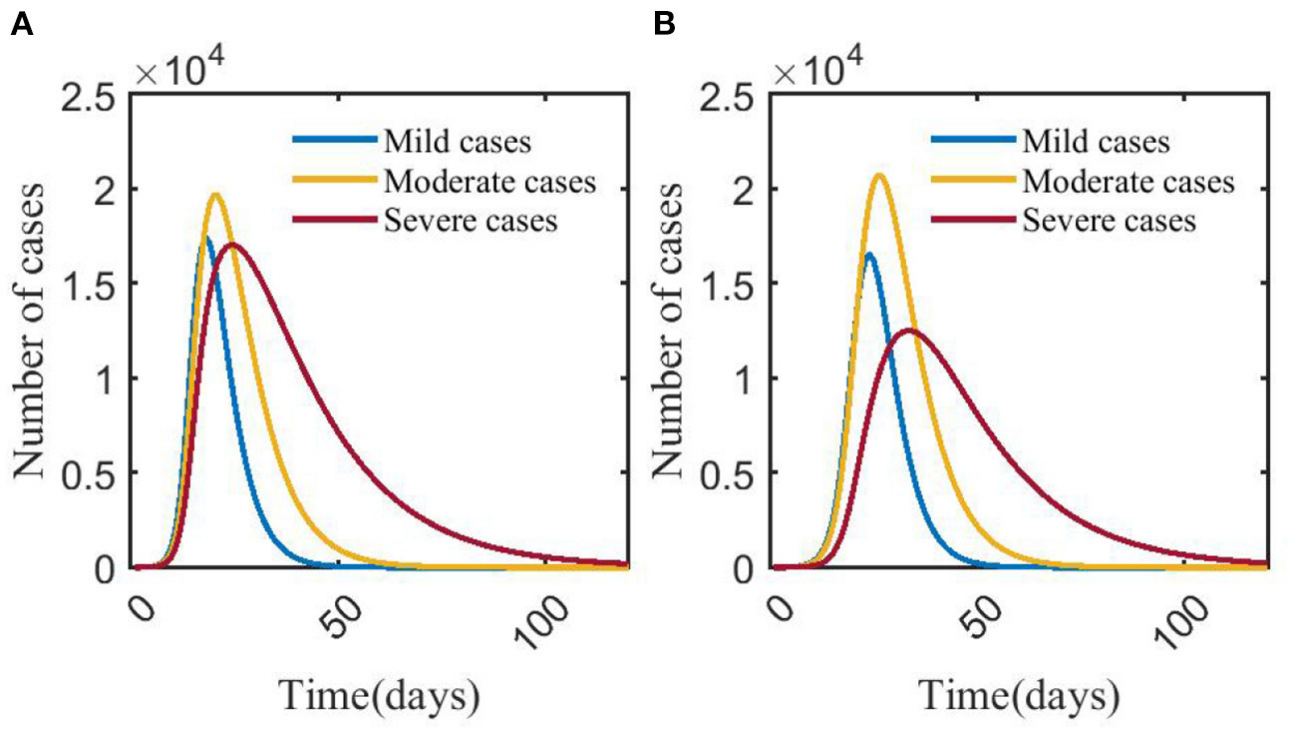
Changes in numbers of mild, moderate, and severe cases in scenarios 1 **(A)** and 2 **(B)**. In scenario 1, we assumed no NPIs or vaccination against COVID-19. In scenario 2, we assumed no NPIs and 33% effectiveness of vaccination to prevent infection of COVID-19. The blue, yellow, and red curves represent the number of mild, moderate, and severe cases, respectively. Scenarios setting are shown in Table 2. Parameter values used are given in Table 3.

Simulation of the model shows that compared with scenario 1 (no NPIs or vaccination), the time for the number of mild, moderate and severe cases to reach the peak in scenario 2 (COVID-19 vaccination has 33% effectiveness for reducing the transmission coefficient of COVID-19) was shortened, and the peak number of mild and severe cases decreased. The number of severe cases in scenario 1 peaked at 17,019 on day 23, and the infections peaked at 51,294 on day 19. Compared with scenario 1, the peak number of severe cases in scenario 2 decreased by 26.57%, the day to reach the peak was delayed by 9 days, and the peak number of infections decreased by 10.16%; the day to reach the peak of infections was delayed by 6 days (Figures 2, **4A**).

### Effects of NPIs on COVID-19—Scenario 3 vs. scenario 4 vs. scenario 5

**Figure 3:**
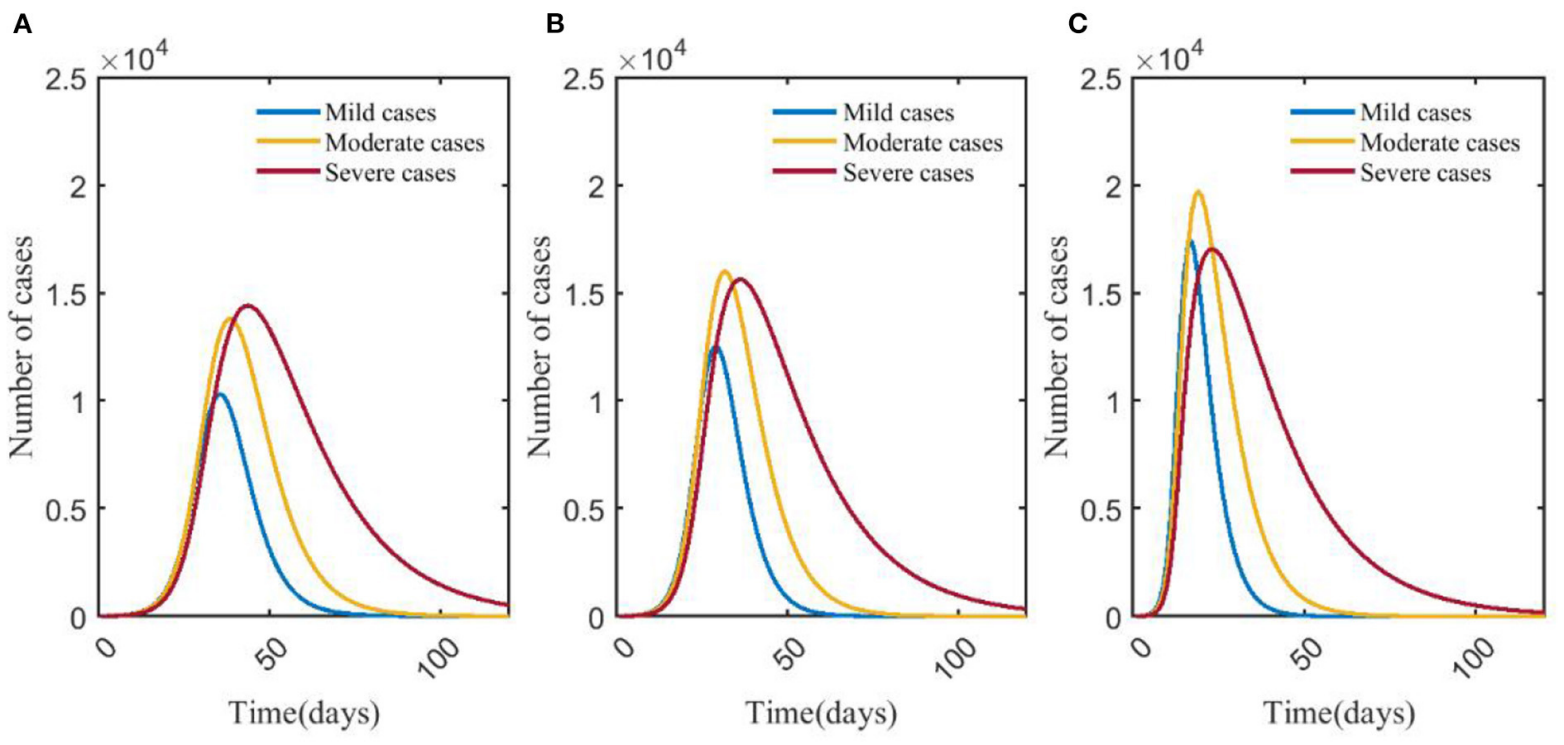
Changes in numbers of mild, moderate, severe cases in scenarios 3 **(A)**, 4 **(B)**, and 5 **(C)**. The blue, yellow, and red curves represent mild, moderate, and severe cases, respectively. Scenarios setting are shown in Table 2. Parameter values used are given in Table 3.

**Figure 4:**
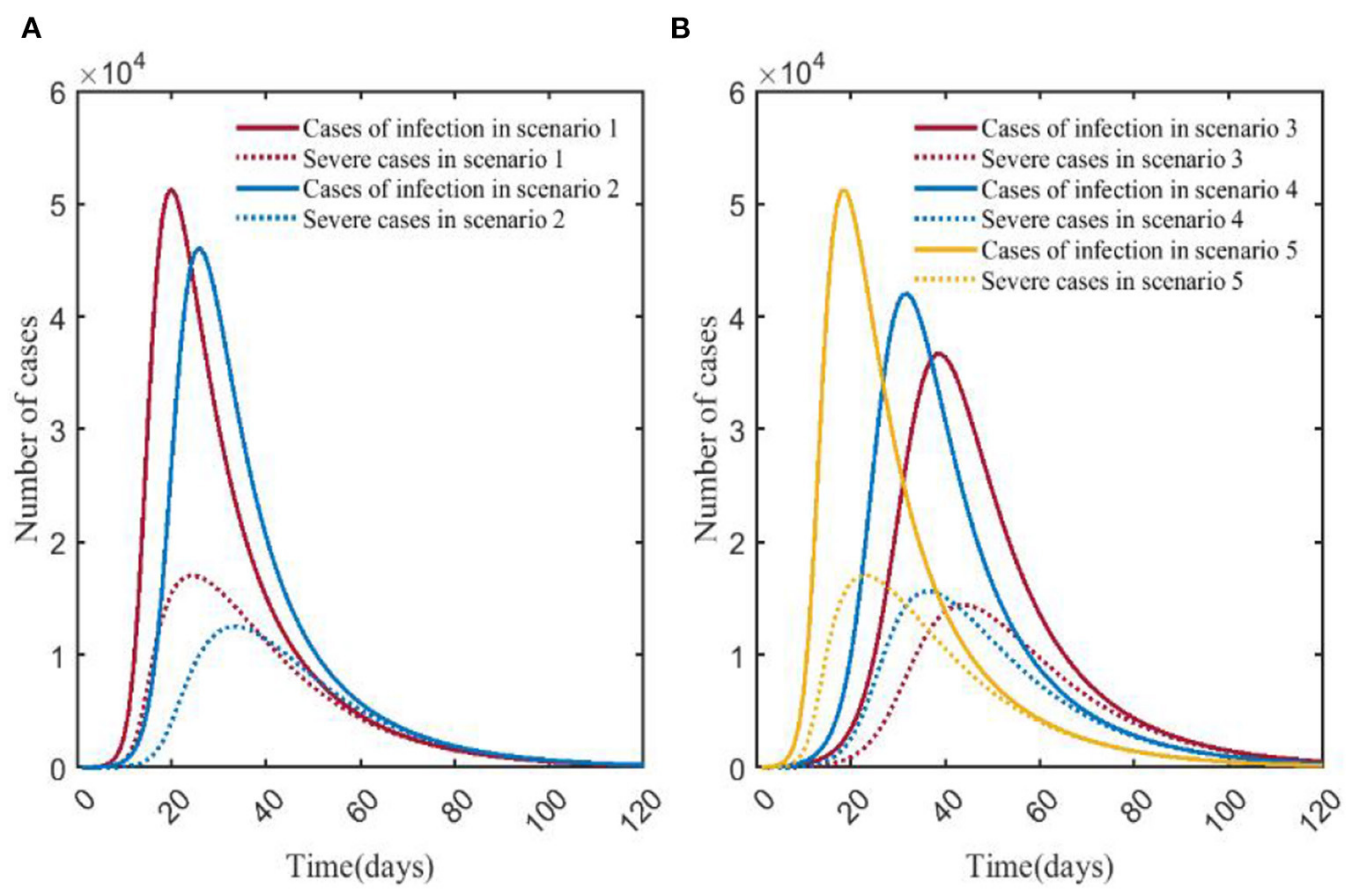
Changes in numbers of severe cases and infections in scenarios 1, 2 **(A)** and scenarios 3, 4, 5 **(B)**. In **(A)**, the red and blue solid curves represent the number of infections in scenarios 1 and 2, respectively. The red and blue dotted curves represent the number of severe cases in scenario 1 and scenario 2, respectively. In **(B)**, the red, blue, and yellow solid curves represent the number of infections in scenario 3, scenario 4, and scenario 5, respectively. The red, blue, and yellow dotted curves represent the number of severe cases in scenario 3, scenario 4, and scenario 5, respectively. Scenarios setting are shown in Table 2. Parameter values used are given in Table 3.

With the strengthening of NPIs, the peak number of mild, moderate, severe, and infections decreased. The peak of severe cases in scenario 3 (14,402) was 7.79% and 15.43% lower than that in scenario 4 (15,620) and scenario 5 (17,031), respectively, and the number of days to reach the peak decreased by 8 and 21 days, respectively. The peak number of infections in scenario 3 (36,695) was 12.67% and 28.28% lower than that in scenario 4 (42,020) and scenario 5 (51,170), respectively. The time to reach the peak number of infections was delayed by 7 and 21 days, respectively (Figures 3, 4B).

### Effects of influenza vaccine—Scenario 6 vs. scenario 7

**Figure 5:**
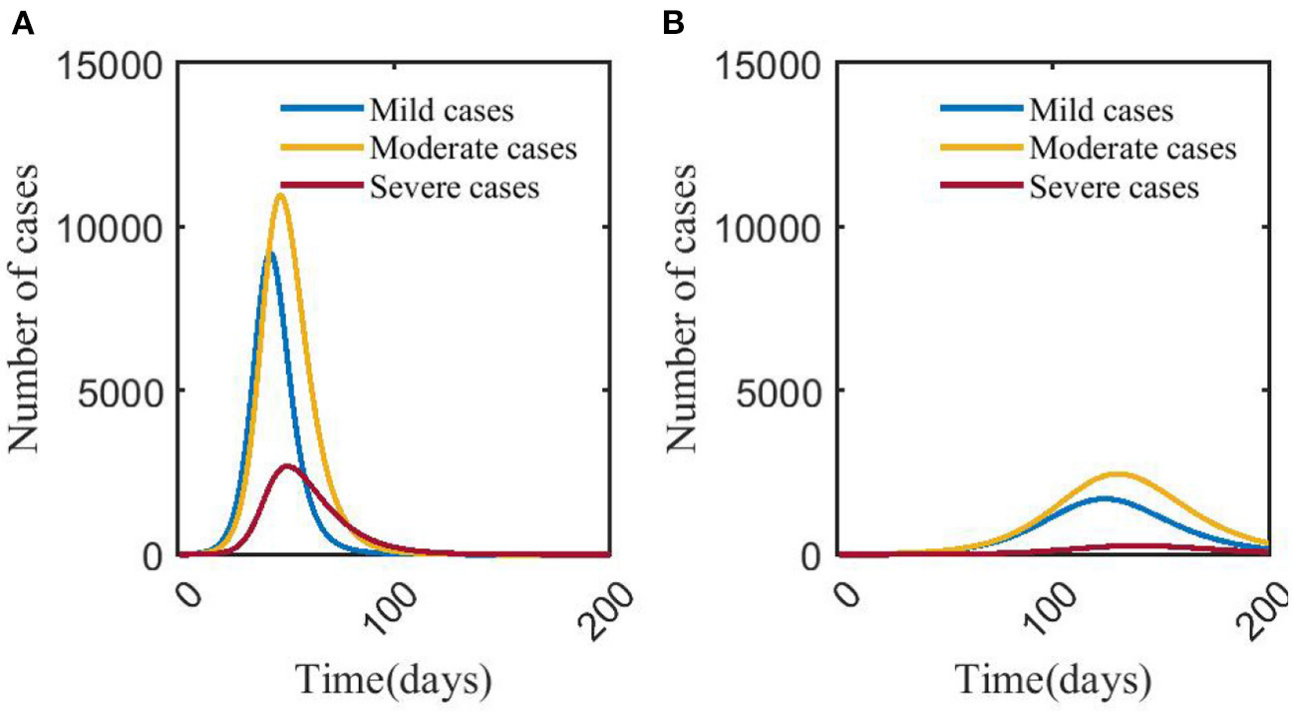
Changes in numbers of mild, moderate, and severe cases in scenarios 6 **(A)** and 7 **(B)**. The blue, yellow, and red solid curves represent the number of mild, moderate, and severe cases, respectively. Scenarios setting are shown in Table 2. Parameter values used are given in Table 4.

It showed that the peak number of severe cases was less than that of mild and moderate cases in scenarios 6 and 7. In scenario 7, the peak number of severe cases was 273, which was 89.85% less than that in scenario 6, and the day to the peak was 88 days later than that in scenario 6. The number of infections in scenario 7 peaked at 4,389, which was 80.01% less than that in scenario 6, and the time to peak was delayed by 83 days (Figures 5, **7A**).

**Table 4:** Parameter combinations of five scenarios of influenza.

**Parameters**	**Scenario**					**Setting basis**
	**Scenario 6**	**Scenario 7**	**Scenario 8**	**Scenario 9**	**Scenario 10**	
*S*	1 × 10^5^	1 × 10^5^	1 × 10^5^	1 × 10^5^	1 × 10^5^	Hypothesis
*E*	20	20	51	51	51	I time the incubation period
*I*_1_	5	5	10	10	10	Hypothesis
*I*_2_	1	1	6	6	6	Hypothesis
*I*_3_	1	1	2	2	2	Hypothesis
*I*	7	7	18	18	18	I_1_ + I_2_ + I_3_
*r*	0	0	0	0	0	Hypothesis
ω	–	–	0.31	0.45	0.53	Reference (7)
β	0.4	0.4 × 0.5	0.4	0.4	0.4	References (9, 19–21)
κ	0.5	0.5	0.5	0.5	0.5	Hypothesis
γ_1_	1/4.67	1/4.67	1/4.67	1/4.67	1/4.67	Reference (22)
γ_2_	1/7	1/7	1/7	1/7	1/7	References (22, 23)
γ_3_	1/14	1/14	1/14	1/14	1/14	Reference (22, 23)
ρ_1_	0.89	0.95	0.89	0.89	0.89	Calculated according to ρ_3_
ρ_2_	0.06	0.04	0.06	0.06	0.06	Calculated according to ρ_3_
ρ_3_	0.05	0.01	0.05	0.05	0.05	Reference (24)
θ_1_	1/4.67	1/4.67	1/4.67	1/4.67	1/4.67	Reference (22)
θ_2_	1/7	1/7	1/7	1/7	1/7	Reference (22)
Ψ	1/2.83	1/2.83	1/2.83	1/2.83	1/2.83	Reference (22)

### Effects of NPIs on influenza—Scenario 8 vs. scenario 9 vs. scenario 10

**Figure 6:**
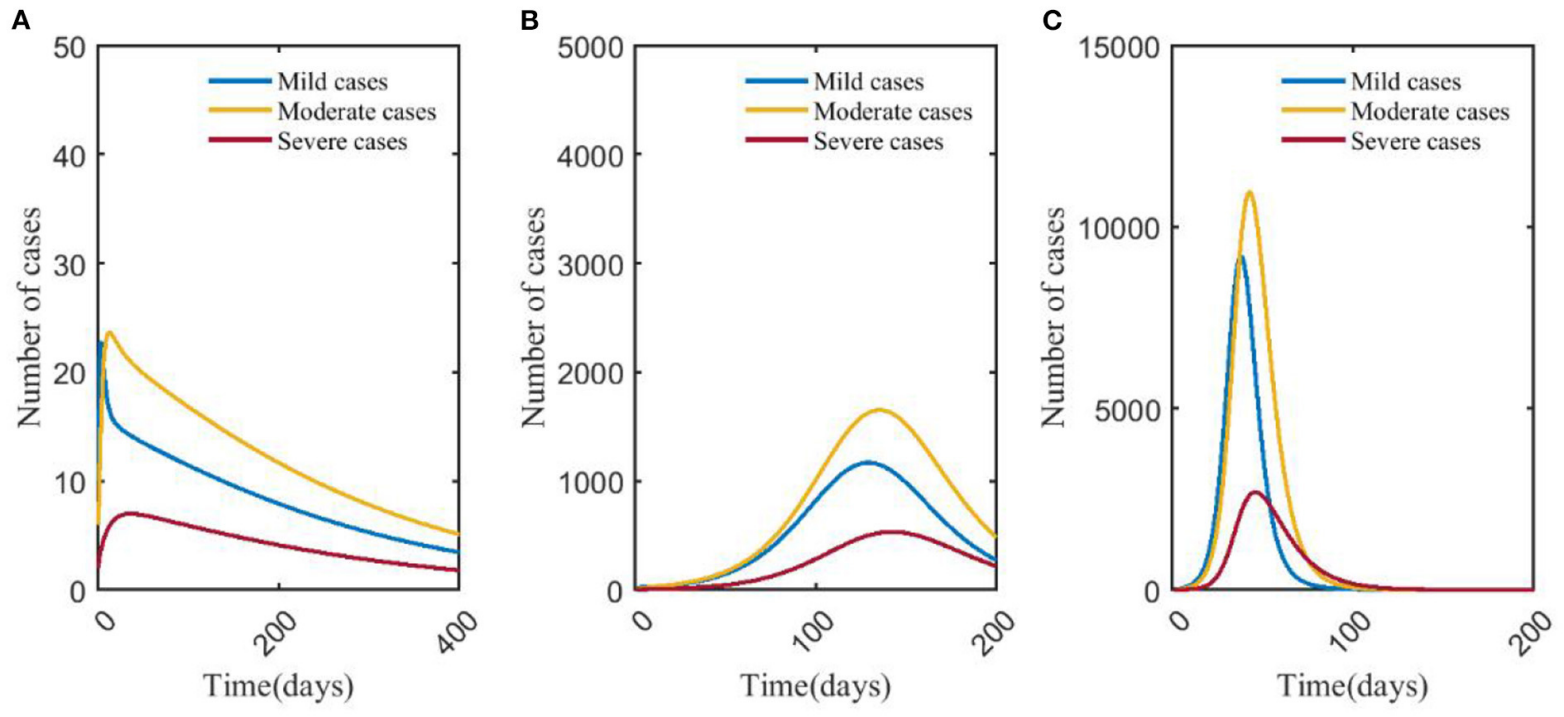
Changes in numbers of cases of different types in scenarios 8 **(A)**, 9 **(B)**, and 10 **(C)**. The blue, yellow, and red curves represent mild, moderate, and severe cases, respectively. Scenarios setting are shown in Table 2. Parameter values used are given in Table 4.

**Figure 7:**
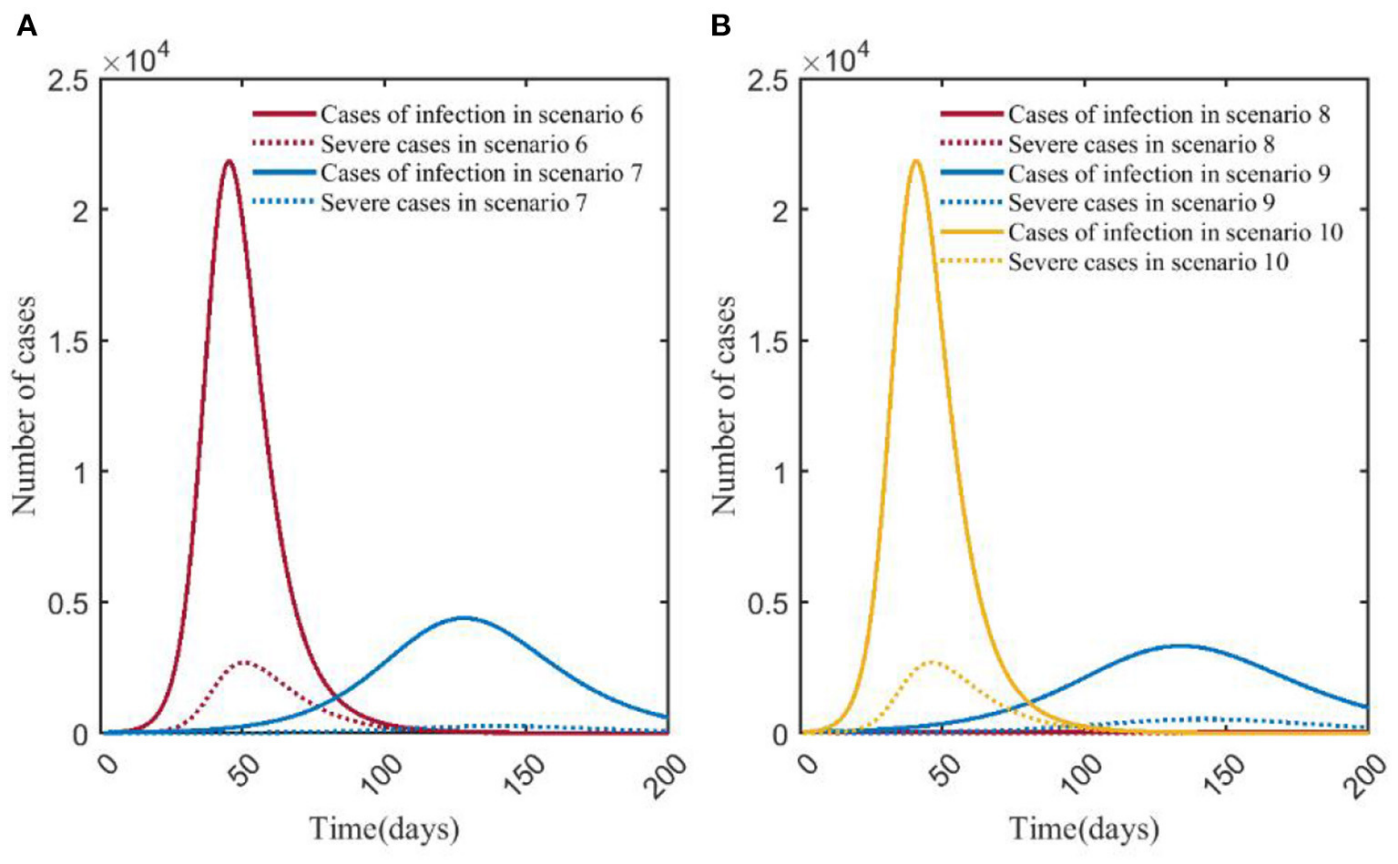
Changes in numbers of severe cases and infections in scenarios 6, 7 **(A)** and 8, 9, 10 **(B)**. In **(A)**, the red and blue solid curves represent the number of infections in scenarios 6 and 7, respectively. The red and blue dotted curves represent the number of severe cases in scenario 6, scenario 7, respectively. In **(B)**, the red, blue, and yellow solid curves represent the number of infections in scenario 8, scenario 9, scenario 10, respectively. The red, blue, and yellow dotted curves represent the number of severe cases in scenario 8, scenario 9, scenario 10, respectively. Scenarios setting are shown in Table 2. Parameter values used are given in Table 4.

Figure 6 showed that after taking different levels of NPIs, the number of severe cases decreased significantly compared with mild and moderate cases. In scenario 8, the number of mild, moderate, and severe cases was <50, and the peak number of severe cases was 7, which was 99.27% lower than that in scenario 9. The time to peak was delayed by more than 3 months. The number of infections (46 cases) peaked on day 9, which was 93.23% lower than scenario 9, and the time to peak was delayed by 124 days. The differences in numbers of severe cases and infections between scenario 8 and scenario 10 could be negligible. The number of severe cases in scenario 10 peaked on day 45, which was about 2,693, and increased by 80.24% compared with scenario 9, and the time to peak was 96 days earlier. The number of infections in scenario 10 peaked on day 40, 93 days earlier than in scenario 9, and the number at its peak increased by 84.77% (Figure 7).

### COVID-19 vs. influenza

**Figure 8:**
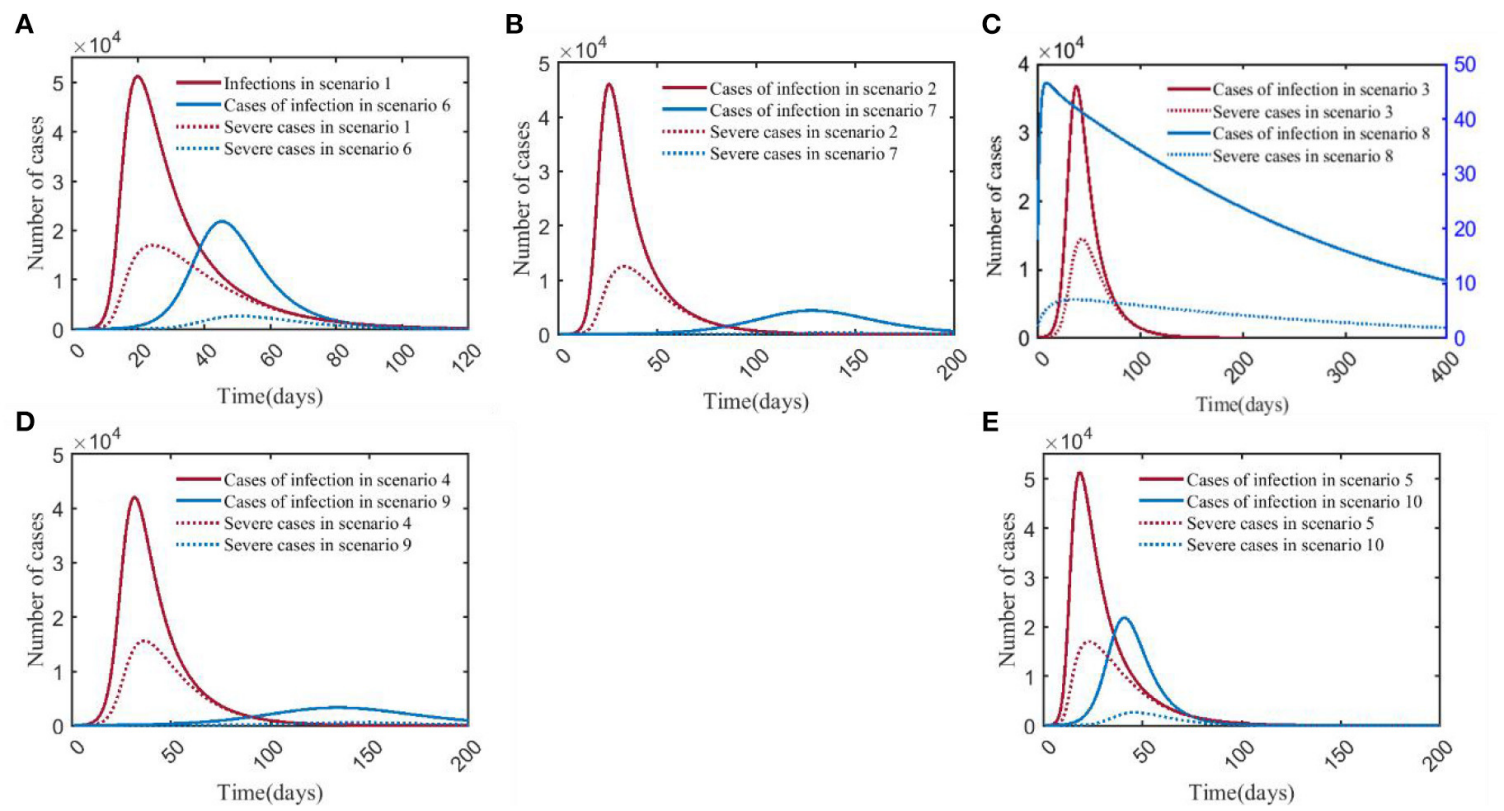
Comparison of numbers of infections and severe cases in scenarios 1–5 and scenarios 6–10. **(A)** is the result of comparison of scenario 1 and scenario 6. **(B)** is the result of comparison of scenario 2 and scenario 7. **(C)** is the result of comparison of scenario 3 and scenario 8 (Applies to the Y-axis on the right side). **(D)** is the result of comparison of scenario 4 and scenario 9. **(E)** is the result of comparison of scenario 5 and scenario 10. The red and blue solid curves represent the number of infections in scenarios 1–10, respectively. The red and blue dotted curves represent the number of severe cases in scenarios 1–10, respectively. Scenarios setting are shown in Table 2. Parameter values used are given in Tables 3, 4.

Scenario 1 and scenario 6 simulated the natural epidemic characteristics of COVID-19 and influenza without taking any measures. The results indicated that the total number of COVID-19 infections peaked on around day 23 (51,294), while influenza peaked on day 50 (21,827). The peak number of COVID-19 infections was more than twice that of influenza. The peak number of severe cases was about 6.32 times than that of the influenza (17,019/2,692), and the time to peak was 27 days earlier (Figure 8A). By comparing scenarios 2 and 7, it showed that the peak of severe cases in scenario 2 appeared on day 32, with 12,497 cases, which was about 45.77 times that in scenario 7 (12,497/273), the number of infections in scenario 2 peaked on day 32, and the peak number was about 10.50 times (46,081/4,389) than that in scenario 7 (Figure 8B). Scenario 3 and scenario 8 were the epidemiological trends of COVID-19 and influenza, assuming that the NPI level was level 3. The results showed that in scenario 8, the peak number of severe cases and infections were both at low levels, with 7 and 46 cases, respectively. In scenario 3, severe cases and infections peaked at 14,402 and 36,695, respectively (Figure 8C). Scenario 4 and scenario 9 simulated the trends of COVID-19 and influenza when the intensity of NPI was level 2. The results showed that the peak number of severe cases of COVID-19 was about 29.39 times than that of influenza (15,620/532), and the time to peak was 106 days earlier; the peak number of COVID-19 infections was about 12.62 times than that of influenza (42,020/3,327), and the time to peak was shortened by 95 days (Figure 8D). Scenarios 5 and 10 assumed that NPI level was level 1. In Figure 8E, it showed that the peak of severe COVID-19 cases was about 6.32 times than that of influenza (17,031/2,693), and the time to peak was 23 days earlier. The peak number of COVID-19 infections was about 2.34 times than that of influenza (51,170/21,859), and the time to peak was 23 days earlier.

**Figure 9:**
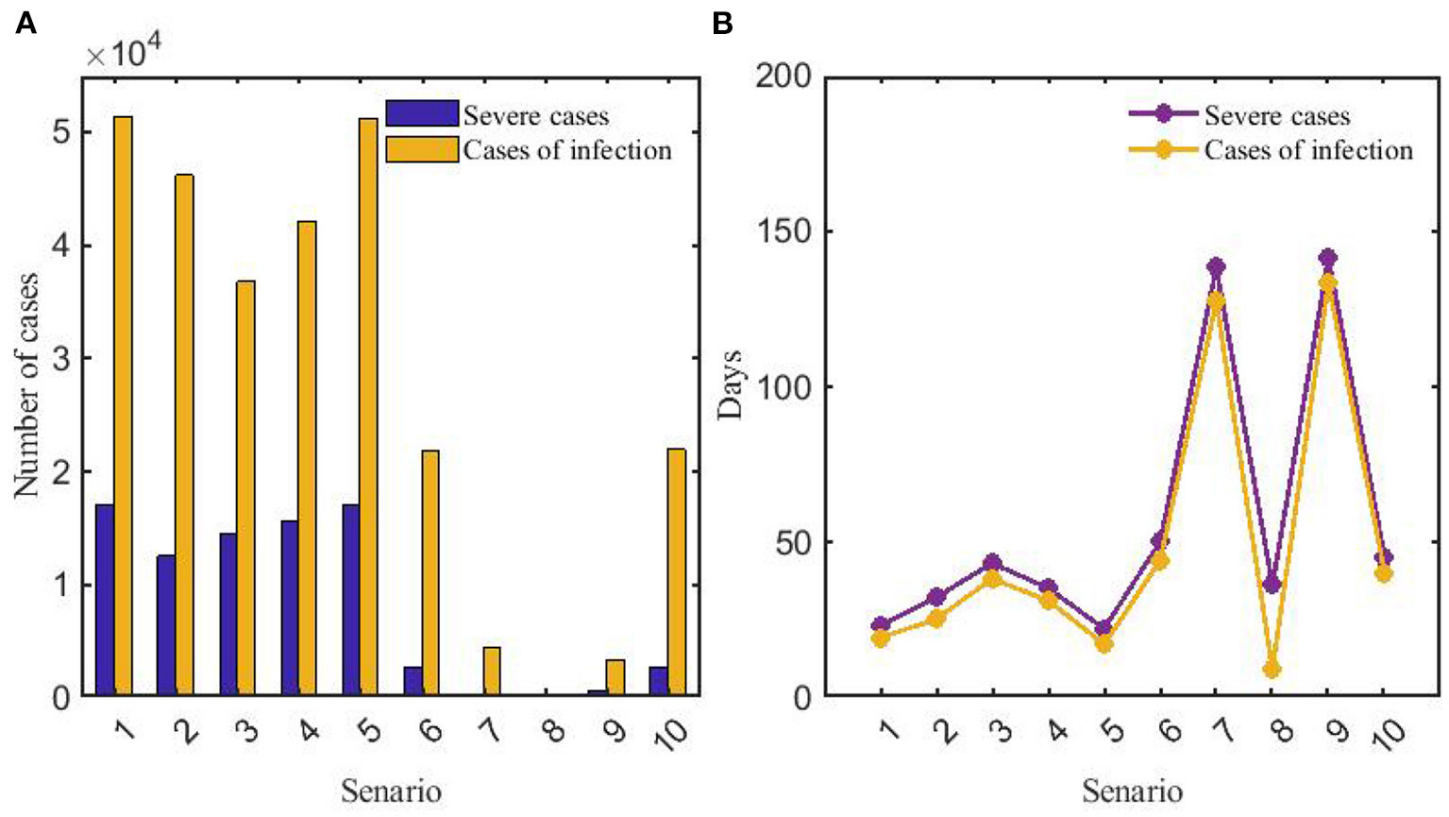
Comparison of peaks **(A)** and time to peak **(B)** of numbers of infections and severe cases in scenarios 1–10. In panel **(A)**, the orange and indigo bars represent the number of infections and severe cases in scenarios 1–10, respectively. In panel **(B)**, the orange and indigo lines represent the time to peak of numbers of infections and severe cases in scenarios 1–10, respectively. Scenarios setting are shown in Table 2. Parameter values used are given in Tables 3, 4.

A horizontal comparison of scenarios 1–10 indicated that the peak numbers of infections and severe cases of COVID-19 were far more than those of influenza pandemic. For COVID-19 (scenarios 1–5), the reduction in the peaks of infections was most significant with the adoption of strict NPIs, and the time to peak could be delayed significantly. We can see that vaccination was the best way to prevent severe COVID-19 cases for the decrease of severe cases in scenario 2. As for influenza (scenarios 6–10), strict NPIs could minimize the peak numbers of both infections and severe cases, and influenza vaccination could significantly delay the time to peak (Figure 9).

## Discussion

In the process of a global response to COVID-19, the prevention and control of influenza still need to be paid enough attention. Fortunately, in the process of responding to COVID-19, more and more people have developed good hygiene habits such as wearing masks and keeping their hands clean, which undoubtedly have positive significance for the prevention and control of influenza. Since severe cases have the greatest demand for medical resources, we focused more on the analysis of the scale of the severe/critically ill population. In order to compare the infections scale of COVID-19 and influenza in the same scenario, we conducted a model analysis on the infections scale and trend of influenza according to the epidemic characteristics of influenza and COVID-19.

### Effectiveness of vaccination on COVID-19 and influenza

Due to differences in national policies, vaccine types, and study samples, the global study results on the effectiveness of COVID-19 vaccines are not uniform. What is certain, however, is that the vaccination effectiveness of coronaVac in preventing infection, morbidity, and hospitalization decreases over time, but by vaccinating a booster dose of coronaVac will increase the neutralizing antibodies and elicit stronger specific immunity than the second dose, today the pandemic is not yet over, and vaccination campaigns are still ongoing, so we chose to set the parameter of vaccine effectiveness to be after three doses of coronaVac. In fact, vaccine effectiveness preventing severe outcomes declines less rapidly than against infection and transmission (25). This is consistent with our findings, but the reduction in the peak number of infections is not as large as in severe cases. Many studies have proven that vaccines are more than 90% effective in preventing severe cases (26–28). At present, as the pandemic continues, many countries have move away from tough prevention and control measures to restrict the movement of people and instead have chosen a more moderate approach to epidemic prevention, that is, coexisting with the virus, thus making the importance of vaccines all the more self-evident. According to the data from WHO, as of 25 July 2022, a total of 12,248,795,623 COVID-19 vaccine doses have been administered (1). In China, as of 20 July, 2022, 92% of the population has been vaccinated with at least one dose of COVID-19 vaccine, 89% of the population has been fully vaccinated, and more than 56% of the population has been vaccinated with at least one booster dose (29). The high vaccination rate and strict NPIs controlled the COVID-19 epidemic at a low level in China. Coronavac is one of the WHO-approved vaccines and over two billion doses have been administered in more than 40 countries. One study showed that SARS-CoV-2 vaccination failed to stop the disease occurrence, but it inhibited the disease severity from mild or moderate to severe or critical (13, 14, 30).

### Effectiveness of NPIs on COVID-19 and influenza

The results indicated that the final scale of both COVID-19 and influenza outbreaks declined significantly as containment efforts intensified. The results of this study showed that vaccination could greatly reduce the peak number of severe COVID-19 cases, and strict NPIs could effectively reduce the peak number of COVID-19 infections. Therefore, we recommend that at the beginning of one pandemic, strict NPIs can be taken to suppress the outbreak quickly, but the economic cost, mental health burden, and excess deaths due to not being able to seek healthcare given strict NPIs should be taken into consideration as well when the government decides to take strict NPIs. We find that influenza vaccination could effectively prevent infectiousness and clinical severity and delay the time to peak of the influenza epidemic. Under the assumption that the effectiveness of NPIs is level 3, the scale of influenza is almost negligible. However, we do not believe that strict NPIs are the most cost-effective method for influenza control in the long time. It is because the model shows that even without NPIs and with only influenza vaccination, the final scale of the influenza epidemic will eventually be within the healthcare system's capacity for most countries and regions.

## Limitations

There are still some limitations to this study. First, scientific decision-making requires reliable evidence support, and the epidemic patterns of diseases should be fully understood. However, due to the complexity of the epidemic in the real world, it is challenging to accurately discover all indicators that impact the epidemic and incorporate them into the model. When evaluating the effect of NPIs, we did not subdivide NPIs and analyze the independent effect of each NPI (such as wearing masks, and maintaining social distancing). Second, the political, economic, cultural, and epidemic situations differ greatly from country to country and region to region, the above influencing factors were not considered in this model, so the real-world situation was not simulated and predicted. Third, we only referred to the effectiveness data of three doses of coronaVac COVID-19 vaccine, regarded the vaccine effectiveness as a constant and did not adjust the model according to the attenuation of vaccine effectiveness. Furthermore, the WHO Strategic Advisory Group of Experts on Immunization (SAGE) recommends that a third, additional dose of the Sinovac vaccine be offered to persons aged 60 and above as part of an extension of the primary series. Current data does not indicate the need for an additional dose in persons under 60 years of age (31), so the study has made overly optimistic vaccination estimates. Finally, the construction of model scenarios is a theoretical analysis. In fact, the effect of epidemic prevention and control is related to the prevention and control capabilities of different regions, and the disease trends may not be the same as predicted in the model.

## Conclusion

The effectiveness of COVID-19 coronaVac vaccine for preventing severe outcomes is better than preventing infection; for the prevention and control of influenza, we recommend influenza vaccination as a priority over strict NPIs in the long term.

## Author contributions

TZ and FL supervised the study. TZ and ZW designed the study. ZW and YC collected data. TZ, ZW, and YC performed analysis. YC, ZW, FL, JM, JZ, YC, and TZ interpreted the findings. YC, ZW, and FL wrote the manuscript. JM, ZW, JZ, YC, and TZ commented on and revised the manuscript accordingly.

## Conflict of interest

The authors declare that the research was conducted in the absence of any commercial or financial relationships that could be construed as a potential conflict of interest.

## Publisher's note

All claims expressed in this article are solely those of the authors and do not necessarily represent those of their affiliated organizations, or those of the publisher, the editors and the reviewers. Any product that may be evaluated in this article, or claim that may be made by its manufacturer, is not guaranteed or endorsed by the publisher.
